# Progress in structural and functional study of the bacterial phenylacetic acid catabolic pathway, its role in pathogenicity and antibiotic resistance

**DOI:** 10.3389/fmicb.2022.964019

**Published:** 2022-09-08

**Authors:** Min Jiao, Wenbo He, Zhenlin Ouyang, Qindong Shi, Yurong Wen

**Affiliations:** ^1^Department of Critical Care Medicine, Center for Microbiome Research of Med-X Institute, The First Affiliated Hospital, Xi’an Jiaotong University, Xi’an, China; ^2^Department of Critical Care Medicine, The First Affiliated Hospital, Xi’an Jiaotong University, Xi’an, China; ^3^The Key Laboratory of Environment and Genes Related to Disease of Ministry of Education Health Science Center, Xi’an Jiaotong University, Xi’an, China

**Keywords:** phenylacetic acid pathway, aromatic metabolites, enzyme structure, pathogenicity, antibiotic resistance

## Abstract

Phenylacetic acid (PAA) is a central intermediate metabolite involved in bacterial degradation of aromatic components. The bacterial PAA pathway mainly contains 12 enzymes and a transcriptional regulator, which are involved in biofilm formation and antimicrobial activity. They are present in approximately 16% of the sequenced bacterial genome. In this review, we have summarized the PAA distribution in microbes, recent structural and functional study progress of the enzyme families of the bacterial PAA pathway, and their role in bacterial pathogenicity and antibiotic resistance. The enzymes of the bacterial PAA pathway have shown potential as an antimicrobial drug target for biotechnological applications in metabolic engineering.

## Introduction

Aromatic hydrocarbons with diverse chemical structures and resistance to degradation are among the most abundant sources of organic carbon in nature (Bugg et al., 2011; Wang et al., 2017). They include plant-soluble secondary metabolic products and structural polymer lignin, some common environmental pollutants, such as petroleum derivatives BTEX (benzene, toluene, ethylbenzene, and xylene), polycyclic aromatic hydrocarbons (PAHs), polychlorinated biphenyls (PCBs), pentachlorophenol (Cao et al., 2009; Fuchs et al., 2011).

Due to limited reactivity, aromatic compounds are predominantly degraded by microbes, which have evolved enzymatic pathways under aerobic and anaerobic conditions (Metzler, 2003; Carmona et al., 2009; Fuchs et al., 2011). The strategy of converging different peripheral pathways by producing a few central intermediates, like phenylacetic acid (PAA), which are then degraded by shared enzymes in subsequent pathways, enables microbes to utilize various aromatic compounds with high efficiency (Fuchs et al., 2011; Liu et al., 2020). Under anaerobic conditions, the intermediate benzoyl-CoA is formed, and two enzymes are responsible for reduction: a class I reductase driven by ATP hydrolysis (Boll and Fuchs, 1995), and a multisubunit enzyme ATP-independent class II benzoyl-CoA reductase (Kung et al., 2009). There are two aerobic strategies to break down aromatic rings: the introduction of two hydroxyl groups into the aromatic ring by a dioxygenase forming catechol, which cleaves the bond adjacent to the carboxyl in an oxygen-dependent manner (Fuchs, 2008); and the attachment of coenzyme A (CoA) to aromatic compounds, which is then catalyzed by a series of monooxygenases (Ismail et al., 2003; Rather et al., 2010; Teufel et al., 2010).

The PAA degradation pathway is the central aromatic compound metabolic pathway utilizing CoA. This pathway contains both aerobic and anaerobic elements and is present in more than 16% of the currently sequenced bacterial genomes (Teufel et al., 2010), as well as in archaea such as *Ferroglobus placidus* (Aklujkar et al., 2014) and Thermoprofundales (Liu et al., 2020). To demonstrate remote evolution and propose the possibility of biotechnological application of this pathway from historical findings as well as recent progress, in this review, we focused on the following aspects of the PAA catabolic pathway: distribution and function of PAA, *paa* cluster components and their structural characteristics, the relationship between PAA metabolic and bacterial pathogenicity, and antimicrobial resistance.

## Phenylacetic acid distribution and function in microbes

PAA is widely distributed across bacteria, fungi, algae, and terrestrial plants. *Pseudomonas* species can use aromatic molecules as the sole carbon source for growth (Dagley, 1975). In other genera such as *Clostridium* (Elsden et al., 1976) and *Bacteroides* (Mayrand, 1979), PAA is as a metabolic product, but its function remains unknown. PAA is a weak acid that is toxic at certain concentrations and pH values, and its wide spread among bacteria and fungi is responsible for deterring non-specific species and preventing habitat loss (Burkhead et al., 1998; Hwang et al., 2001; Kim et al., 2004a,b; Somers et al., 2005). In *Pseudomonas aeruginosa*, 1.47 mM PAA disrupts quorum sensing and attenuates biofilm formation (Musthafa et al., 2012). PAA from the soil bacterium *Bacillus licheniformis* is effective against *Staphylococcus aureus* and *Escherichia coli* (Kim et al., 2004a,b). In fungi, PAA is a direct precursor for penicillin G (Moyer and Coghill, 1947), and affects metabolism at the transcriptional and protein levels (Harris et al., 2009; Jami et al., 2018). Based on the application of the above findings, genetic manipulation to decrease PAA degradation achieved penicillin overproduction in *P. chyrysogeneum* (Rodríguez-Sáiz et al., 2001, 2005). As a ubiquitous plant auxin, PAA promotes plant growth, specifically cell expansion, elongation and differentiation, cubature, callus growth, and lateral root induction, as well as antimicrobial activity (Cook, 2019). The PAA catabolic pathway has not been detected in plants, and animals have only limited capacity to metabolize aromatic compounds (Metzler, 2003).

Microbes including bacteria and archaea are the main organisms in aromatic compound metabolism, suggesting a remote origin of the PAA pathway (Metzler, 2003). In addition to the numerous studies in bacteria, an increasing number of studies in archaea have also demonstrated that they exploit aromatic compounds as energy and carbon sources (Erdoğmuş et al., 2013; Aklujkar et al., 2014). Halophilic archaea use dioxygenases, while hyperthermophilic archaea use the BCoA pathway to mineralize aromatic compounds (Fairley et al., 2006; Schmid et al., 2015). Genomic and biochemical evidence shows that Thermoprofundales can utilize aromatic compounds through the PAA pathway under extreme conditions, such as pH 5–7, and relatively high optimal temperatures (Liu et al., 2020), which could enable application of PAA pathway-related enzymes in biotechnology.

## Protein structural and functional study in PAA pathway

**Figure 1:**
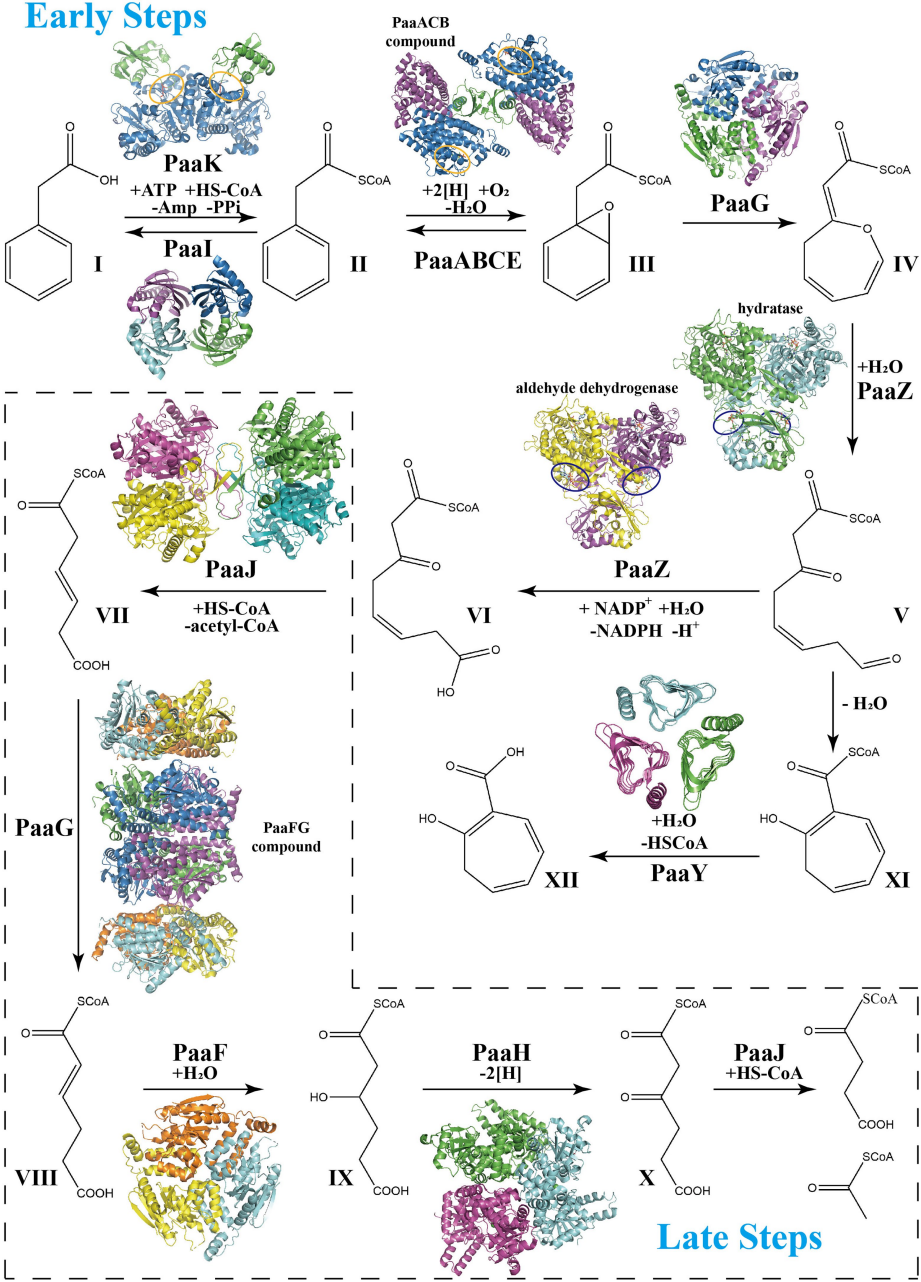
Schema of phenylacetate acid degradation pathway. Early PAA pathway: Step 1, Phenylacetate (**I**, PA) is converted into phenylacetyl-CoA (**II**, PA-CoA), catalyzed by a phenylacetate-CoA ligase PaaK. Thioesterase PaaI could lead this step into a reversible direction when toxic accumulation occurs. Step 2, Epoxidation induced by the monooxygenase complex PaaABCDE in ring 1,2-epoxyphenylacetyl-CoA (**III**, ep-CoA). Step 3, PaaG isomerize ep-CoA into 2-oxepin-2(3H)-ylideneacetyl-CoA (**IV**, oxepin-CoA), an oxygen-containing heterocycle with three double bonds. Step 4, hydrolysis mediated by the bifunctional enzyme PaaZ induces the oxepin-CoA ring-opening and conversion into 3-oxo-5,6-dehydrosuberyl-CoA semialdehyde (**V**). Compound V spontaneously rearranges to 2-hydroxycyclohepta-1,4,6-triene-1-formyl-CoA (**XI**), which inhibits the enzymatic activity of PaaZ. Thioesterase PaaY could relieve this inhibition by converting XI into 2-hydroxycyclohepta-1,4,6-triene (**XII**). Step 5, PaaZ also induces the oxidation of the terminal aldehyde group in V, finally producing 3-oxo-5,6-dehydrosuberyl-CoA (**VI**). Late PAA pathway: Step 6, Thiolase PaaJ induces degradation of long-chain intermediates VI into a C_6_-intermediate, 3,4-dehydroadipyl-CoA (**VII**). Step 7, The isomerization of converting VII into 2,3-dehydroadipyl-CoA (**VIII**) also mediated by isomerase PaaG. Step 8, Hydratase PaaF generates 3-hydroxyadipyl-CoA (**IX**) from VIII. PaaF could form stable complex with PaaG, which may speed up Step 7 and 8 *in vivo*. Step 9, Dehydrogenase PaaH oxidizes IX to 3-oxoadipyl-CoA (**X**). Step 10, The last step of the PAA pathway, degradation of the C_6_-intermediate into succinyl-CoA and acetyl-CoA, also induced by thiolase PaaJ. The structural representations of the key enzymes in the PAA pathway are also shown in corresponding catalyzing steps. The repressors PaaX and PaaR are not shown.

The gene cluster associated with PAA catabolism is the paa operon, which mainly encodes 12 enzymes or enzymatic subunits: PaaZ, PaaA, PaaB, PaaC, PaaE, PaaF, PaaG, PaaH, PaaJ, PaaK, PaaY, and PaaI; transcription regulator PaaX or PaaR in different species; and PaaD protein with unknown function. The PAA pathway can be artificially divided into two parts, the early and late steps (Figure 1), which are similar to that of the benzoate degradation pathway and fatty acid β-oxidation process, respectively. With XI being the precursor for tropone natural products, a recent review has highlighted the structure features of the main enzymes in PAA catabolic pathway to form XI (Grishin and Cygler, 2015; Duan et al., 2020), while we focus on the entire PAA catabolic process. The overview of **t**he catalytic properties of some enzymes are listed in Table 1.

**Table 1:** Overview of the catalytic properties of PAA pathway.

Protein	Organism	Substrate	Temperature*/*°C	Km/μM	Kcat/min^−1^	Vmax/Umg^−1^	References
BcePaaK1	*Burkholderia cenocepacia*	Phenylacetic acid		62	250		Law and Boulanger, 2011
BcePaaK2				150	300		
TthPaaK	*Thermus thermophilus*	Phenylacetate	75	6	1,200	24	Erb et al., 2008
		CoA		30	1,200		
		ATP		50	1,200		
Py2PaaABCE	*Pseudomonas* sp. Y2	Phenylacetyl-CoA	30	1			Teufel et al., 2010
		NADPH		23		1	Teufel et al., 2012
		NADH				0.05	
		Phenylacetyl-CoA		6			
		O2		3			
		Epoxyphenylacety-CoA		17			
TthPaaG	*Thermus thermophilus*	1,2-epoxyphenyl- acetyl-CoA				138	Spieker et al., 2019
Py2PaaG	*Pseudomonas* sp. Y2					182	
		Trans-3,4-didehydroadi poyl-CoA		564	0.21		
TthPaaG	*Thermus thermophilus*			142	3.6		
Py2PaaZ (hydrolysis)	*Pseudomonas* sp. Y2	Oxepin-CoA	22	35		7.6	Teufel et al., 2011, 2012
	*Escherichia coli*						
EcoPaaZ (dehydrogenase)				20		33	Teufel et al., 2011
				11		32	
		NADP+		56			
Py2PaaI	*Pseudomonas sp.* Y2	Phenylacetyl-CoA	22			1.6	Teufel et al., 2012
EcoPaaI	*Escherichia coli*						
AevPaaI	*Aromatoleum evansii* DSM6898		25	9.6	24.6		Song et al., 2006
			25	390	222		
SphPaaI	*Streptococcus pheumoniae*	Phenylacetyl-CoA		90	390		Khandokar et al., 2016
		Decanoyl/C10-CoA		183	1968		
Py2PaaY	*Pseudomonas sp.* Y2	2-hydroxycyclohepta- 1,4,6-triene-1- carboxyl-CoA	22	35		7.6	Teufel et al., 2012
TthPaaR1	*Thermus thermophilus*			1.1 × 10^−3^	6 × 10^−2^		Sakamoto et al., 2011
TthPaaR2				9 × 10^−2^	5.4 × 10^−2^		

### Early steps

#### Activation of the aromatic compound

The phenylacetate-CoA ligase PaaK acts as the initial enzyme in the PAA pathway, controlling the influx of the substrate phenylacetic acid. CoA is attached to phenylacetate by PaaK, which is dependent on Mg^2+^ and ATP, with high specificity and relatively high heat stability (El-Said Mohamed, 2000; Erb et al., 2008).

**Figure 2:**
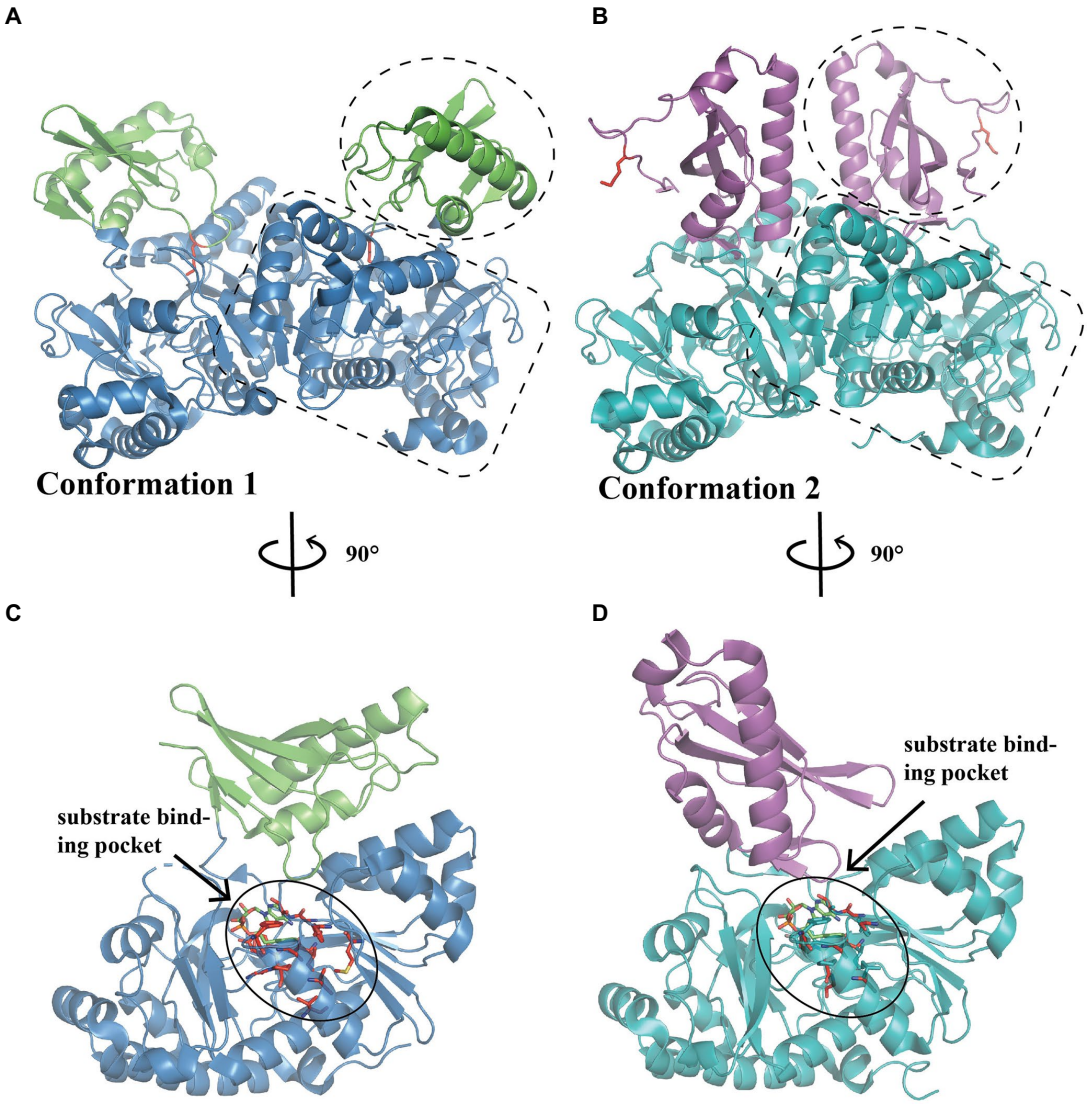
Overall structure of PaaK. **(A)** The overall structure of dimer BcePaaK1 (PDB: 2Y4N) in conformation 1. The N-terminal domain and C-terminal domain are circled by a dotted line in blue and green, respectively, the Lys422 at the active site is shown as sticks in the side chain in red. **(B)** The overall structure of dimer BcePaaK2 (PDB: 2Y4O) in conformation 2 with a similar N-terminal domain and a rotated C-terminal domain. The N-terminal domain and C-terminal domain are circled as in **(A)** in teal and magenta, respectively. The corresponding Lys429 is shown as in **(A)**. The PaaK co-structures with phenylacetyl-adenylate are captured in alternate conformations **(C**,**D)**. The residues involved in the binding pocket are shown as sticks in red and substrates are shown as sticks in green.

PaaK belongs to the adenylate-forming enzyme superfamily and functions as an activator of short-to-long fatty acids, aromatic compounds, and biosynthesis of peptide antibiotics and siderophores (Gulick, 2009). This family member exhibits two α/β domains, with an active site located at the interface between the N-terminal and C-terminal domains (Figures 2A–D; Gulick, 2009). A dimer is mainly formed and maintained by residues from the N-terminal domain, leaving the C-terminal domain free for conformational changes during adenylation catalysis (Law and Boulanger, 2011). Law et al. characterized the structure of two BcePaaK proteins from *Burkholderia cenocepacia*, BcePaaK1 (PDB:2Y4N) and BcePaaK2 (PDB:2Y4O), providing a comprehensive view of PaaK conformation. Briefly, BcePaaK1 catalyzes the adenylation reaction in which lysine (Lys422) at the active site is essential for substrate binding and nucleophilic attack (Figures 2A,C). Conversely, BcePaaK2 exhibits a different conformation during catalysis. In BcePaaK2, the C-terminal domain rotation removes the equivalent lysine (Lys429) from the active site as in BcePaaK1 and provides an alternative platform for the thioesterification reaction (Figures 2B,D).

When the native phenylacetic acid substrate was bound, the paralogs displayed a difference in kinetics. BcePaaK1 showed a lower *K_m_* than BcePaaK2 (62 μM vs.150 μM, respectively), but a similar *K_cat_* (250 min^−1^ and 300 min^−1^ for BcePaaK1 and BcePaaK2, respectively), which may be caused by a deeper aryl binding pocket (Figures 2C,D; Law and Boulanger, 2011). In addition, BcePaaK1 displayed wider substrate specificity than BcePaaK2 for phenylacetic acid derivatives. In *Thermus thermophilus, V_max_* of TthPaaK was 24 U/mg under the most active reaction conditions at a pH of 7.5–8.0 and temperature of 75°C. The *K_cat_* was 20 s^−1^ per subunit, and *K_m_* was 6, 30, and 50 μM for phenylacetate, CoA, and ATP, respectively. Mg^2+^ is required for enzymatic reactions and can be substituted by Mn^2+^ with 90% activity (Erb et al., 2008).

#### Epoxidation of the aromatic ring

The oxygenase complex catalyzes the most crucial step of the pathway, comprising PaaA, B, C, D, and E, inducing oxygen into the aromatic ring of II (Fernández et al., 2006). Through co-expression and pull-down assay of each component, PaaAC, PaaBC, and PaaABC were confirmed to form stable complexes, while PaaD and PaaE could not be detected (Teufel et al., 2010; Grishin et al., 2011). Further *in vitro* reconstitution experiments demonstrated that PaaA, B, C, and E subunits are required for the oxidation reaction and formation of epoxide (Grishin et al., 2011). The reductase subunit PaaE, catalytic subunit PaaA, structural subunit PaaC, and bridging subunit PaaB form the overall complex composition PaaA_2_B_3-4_C_2_E_1_, which contains six iron atoms, two of which belong to the iron–sulfur cluster of PaaE and four to the two molecules of PaaA (Teufel et al., 2012). In their studies, a model for PaaABCE catalysis and catalytic di-iron center-state conversion was proposed. First, the ground-state di-iron core was reduced in an NADPH-dependent manner. The reduced diferric compound could then interact with oxygen, producing a high-valent intermediate or alternatively abstracting the epoxy-oxygen to reverse the reaction. Finally, the high-valent intermediate oxidate II produced III and a ground state di-iron core (Teufel et al., 2012).

**Figure 3:**
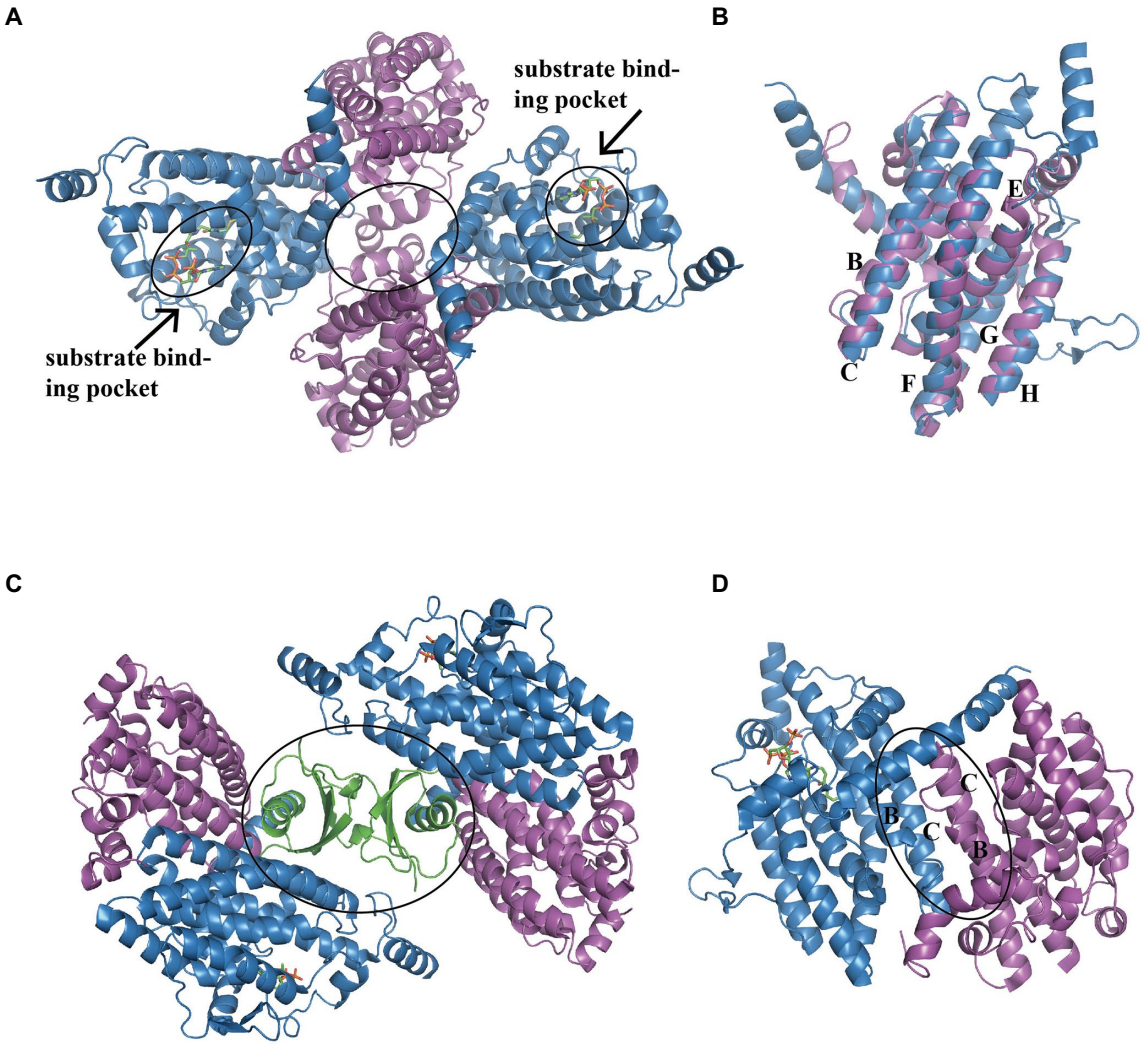
Overall structure of the PaaACB complex. **(A)** The overall structure of heterotetramer EcoPaaAC (PDB:3PW8), the substrate acetyl-CoA in binding pocket is shown as sticks, the complex is formed by interactions in PaaC, circled with a solid line. **(B)** Structure superposition of PaaA and PaaC; the core helixes are labeled as reported previously (Grishin et al., 2011). **(C)** The PaaAC heterodimer formed by interactions between helixes B and C in each subunit. **(D)** The (PaaACB)_2_ heterohexamer from *Klebsiella pneumoniae* (PDB:4IIt) maintained by the dimerization of PaaB, constructing a β-barrel in the center part. PaaA, PaaC, and PaaB are in blue, magenta, and green, respectively.

In *E. coli*, the heterotetramer PaaAC (PDB:3PW8) is similar to PaaA and PaaC subunits (Figure 3A; Grishin et al., 2011). Briefly, the core consisted of six long α-helices: B, C, E, F, G, and H, as previously reported (Grishin et al., 2011; Figure 3B). Helices B and C from each subunit form an antiparallel four-helix bundle that is involved in heterodimer oligomerization (Figure 3C). The substrate binds only to the PaaA subunit, located in a tunnel extending from the protein surface toward the center. The structure of the PaaACB complex from *Klebsiella pneumonia* (PDB:4IIt) reveals a 2:2:2 combination ratio, and the heterohexameric PaaACB is more likely in a PaaAC-(PaaB)_2_-PaaAC pattern (Grishin et al., 2013). In this model, the PaaB dimer was located in the middle part, forming a plateau for the two PaaAC heterodimer assemblies (Figure 3D; Grishin et al., 2013). The PaaAC subcomplex showed the same conformation as the heterodimer, with the PaaB subunit binding to both PaaA and PaaC subunits in a cleft near the PaaA/PaaC interface (Figure 3D).

The end-products measured by LC–MS demonstrated that PaaA, B, C, and E subunits constitute the optimum reaction mixture, while the PaaD subunit showed no effect on the reaction *in vitro* (Teufel et al., 2010; Grishin et al., 2011). However, previous *E. coli* knockout mutant studies indicated that PaaD is essential for this reaction *in vivo*, suggesting that PaaD may induce maturation of the monooxygenase complex, rather than have a direct involvement in catalysis. In addition, PaaB is important for product concentration, which shows a more than 100-fold reduction in its absence (Grishin et al., 2011).

**Figure 4:**
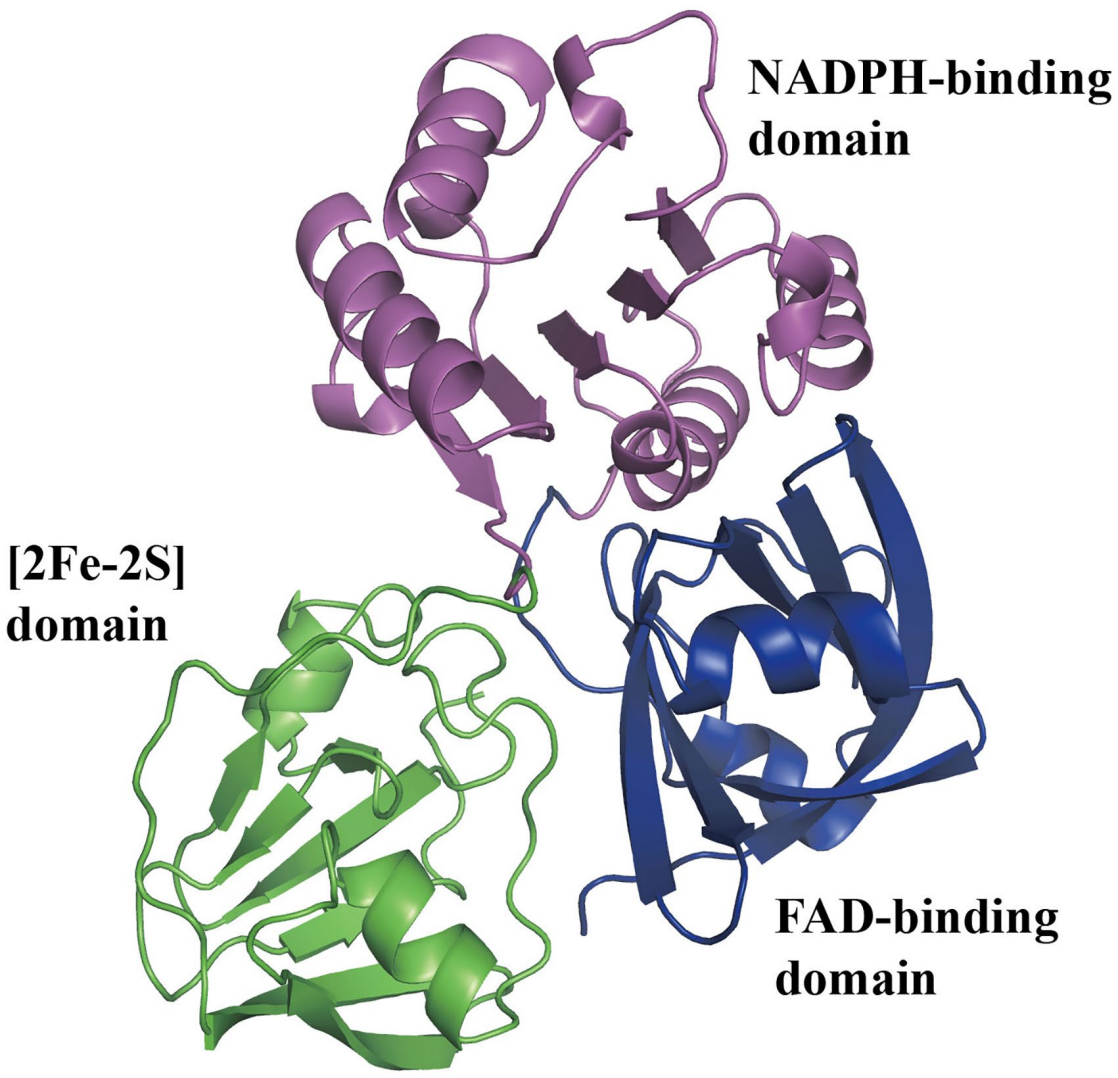
Predicted structure of PaaE. The overall predicted structure of EcoPaaE from AlphaFold. The PaaE is formed by three domains individually, N-terminal FAD-binding domain, NADPH-binding domain, and C-terminal [2Fe-2S] ferredoxin-like domain, shown in blue, magenta, and green, respectively.

Using ^13^C-labeled substrates and detecting by ^13^C-NMR spectroscopy, the Py2PaaABCE complex catalyzed the reaction induced by the compound II, mediating NADPH consumption and depending strictly on oxygen with approximately 1 μmol min^−1^ mg^−1^ protein (Teufel et al., 2010). In another study, the features of PaaABCE were well described (Teufel et al., 2012). Under optimum conditions of 30°C and pH 8.0, epoxidase compound activity was approximately 1.0 U mg^−1^ with NADPH and 0.05 U mg^−1^ with NADH, and apparent *K_m_* were 23 μM, 6 μM, 3 μM, and 17 μM for NADPH, the compound II, O_2_, and the compound III, respectively. PaaE belongs to the class IA reductases, associated with dioxygenases, with an N-terminal NADPH- and FAD-binding domain and a C-terminal [2Fe-2S] ferredoxin-like domain (Figure 4). The absorption spectrum of MBP-EcoPaaE showed that the spectrum maxima closely resemble those of spinach [2Fe-2S] ferredoxin (Grishin et al., 2011). PaaE is thought to transfer electrons from NADPH through FAD and the iron–sulfur cluster to the iron atoms in the active center of PaaA. The oxidoreductase activity of SpePaaE was demonstrated by the nitroblue tetrazolium (NBT) reduction assay, where NBT serves as an electron acceptor and specifically utilizes NADH to transfer electrons. In addition, flavin obtained from heat-denatured PaaE and confirmed by HPLC provided evidence of the presence of the FAD-binding domain (Niraula et al., 2010). None of the structures of PaaE are available. The phthalate dioxygenase reductase from *Burkholderia cepacia* (PDB code 2PIA, Correll et al., 1992) is a structural homolog with 22% sequence identity.

#### Isomerization mediated C–C bond cleavage

After the compound III (Figure 1) is produced during the monooxygenation of the aromatic ring, PaaG isomerizes and finally introduces an α,β-unsaturated CoA-thioester motif. PaaG belongs to the enoyl-CoA hydratase/isomerase family, together with PaaF (late steps). These family members strictly depend on CoA-thioester substrates because of the negatively charged transition state in the form of thioester enolate, stabilized by a dedicated pocket at the active site (Spieker et al., 2019). To evaluate the kinetic parameters of Py2PaaG and TthPaaG, substrates at different catalytic steps were identified using HPLC, spectrophotometry, or UPLC–MS. TthPaaG showed a specific activity of 138 U mg^−1^ in isomerizing the compound III in the early steps of PAA pathway and 182 U mg^−1^ for Py2PaaG. As native cis-VII is unstable, the more stable isomer, trans-VII, which could be converted to the same product, was used to evaluate PaaG activity in the late PAA pathway. The specific activity was 7.6 × 10^−3^ and 116, *K_m_* was 564 and 142 μM, *K_cat_* was 3.5 × 10^−3^ and 60 × 10^−3^ s^−1^, and catalytic efficiencies in Py2PaaG and TthPaaG were 6 × 10^−6^ and 4 × 10^−4^ s^−1^ μM^−1^, respectively (Spieker et al., 2019). Additionally, an aspartate side chain (D136) at the catalytic site of *Pseudomonas* sp. Y2 PaaG acts as a proton relay amino acid and substantially affects enzyme functionality in mutants (Spieker et al., 2019).

**Figure 5:**
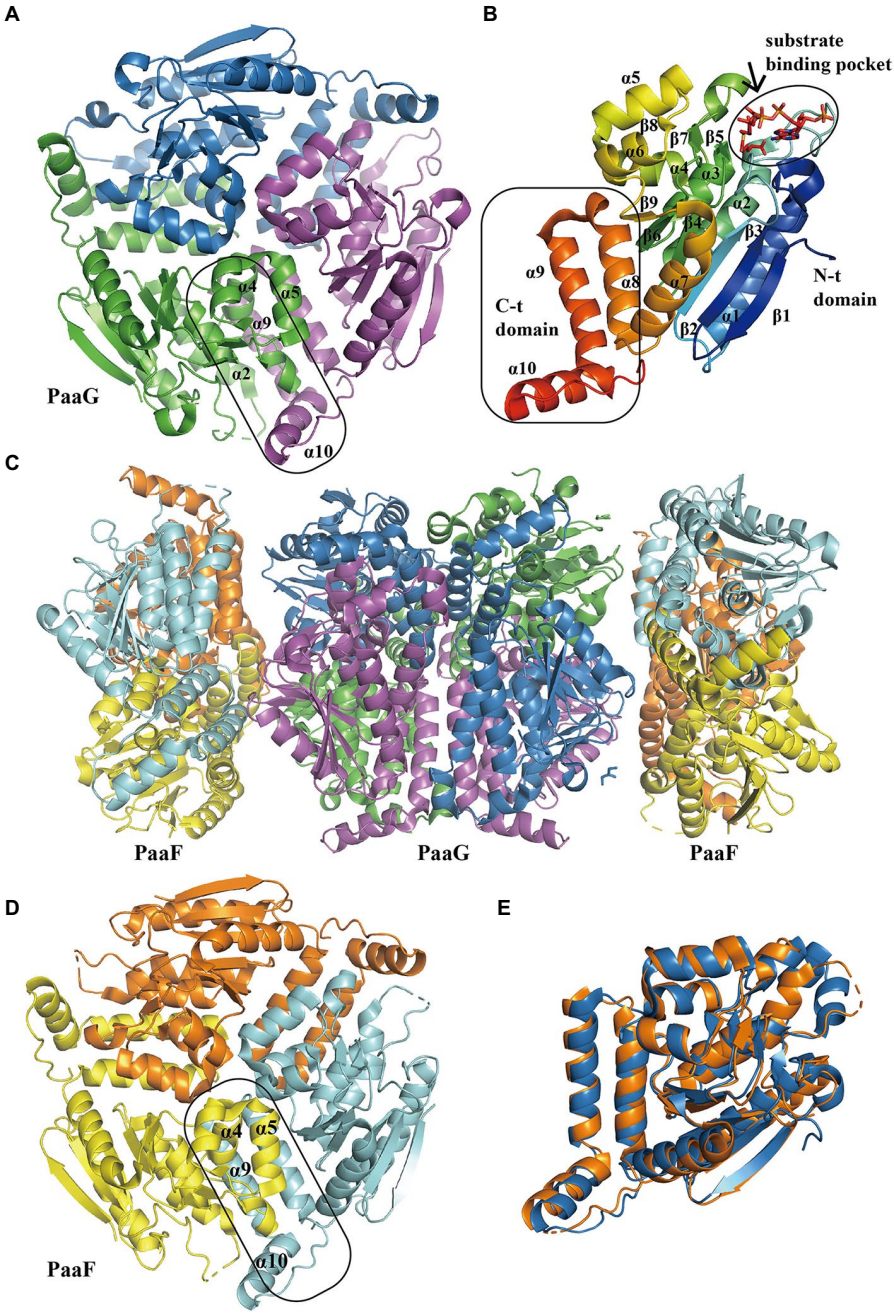
Overall structure of PaaG and PaaF. **(A)** The overall structure of trimer TthPaaG (PDB:6SLB). The interface is circled with a solid line. The different chains are shown in blue, violet, and green, respectively. **(B)** The secondary structure elements in PaaG monomer with the substrate trans-3,4-didehydroadipyl-CoA binding in N-spiral domain. The N-terminal and C- terminal domains are circled with a solid line. The monomer is colored as the rainbow and the substrate is shown as sticks. **(C)** The overall structure of EcoPaaFG complex (PDB:4FZW). The dimer of trimer PaaG is covered with two PaaF trimers. PaaF chains are shown in orange, yellow, and cyan, and PaaG is shown in the same colors as in **(A)**. **(D)** The overall structure of trimer PaaF, reveals a similar shape and interaction site as PaaG trimer as PaaG. **(E)** The structure alignment between PaaF and PaaG monomer reveals a similar crotonase fold feature.

The structure of PaaG reveals the fold features of the enoyl-CoA hydratase/isomerase (crotonase) superfamily. The first report of PaaG structure was based on *Thermus thermophilus* in 2009 (Kichise et al., 2009). A recent study revealed the isomerase TthPaaG in complex with its native ligands, providing a comprehensive view of PaaG-isomerizing substrates in different steps. The disk-shaped trimer PaaG is related to local three-fold symmetry (Figure 5A; Spieker et al., 2019). During the interaction with different ligands, the adenine moiety in CoA is located at the bottom of an open binding pocket, and the acyl moiety of the ligands is oriented toward a shallow hydrophobic site (Figure 5B).

#### Hydrolysis mediated C–O heterocycle cleavage and ring opening

In the next two steps, the bifunctional enzyme PaaZ catalyzes the hydrolysis of the compound IV to V (Figure 1), and oxidation of the terminal aldehyde group, finally producing the compound VI. Previous studies have shown that EcoPaaZ is crucial for the removal of toxic intermediates in the early steps of the PAA pathway, with a specific activity of approximately 20 μmol min^−1^ mg^−1^ protein in the compound IV ring cleavage (Teufel et al., 2011). A subsequent study determined the rate of PaaZ-catalyzed hydrolysis to be 33 U mg^−1^ *via* a photometric test (Teufel et al., 2011). They also reported that the proposed open-chain aldehyde, after hydrolytic ring fission, could rapidly form a seven-membered carbon ring through Knoevenagel-type condensation and finally rearrange into a more stable enol-form compound, inhibiting PaaZ activity (Teufel et al., 2011). In addition, the dehydrogenase activity was approximately 32 U mg^−1^ by measuring NADPH formation using a photometric assay. The *K_m_* was 11 μM and 56 μM for catalyzing the compound IV and NADP^+^ respectively (Teufel et al., 2011).

**Figure 6:**
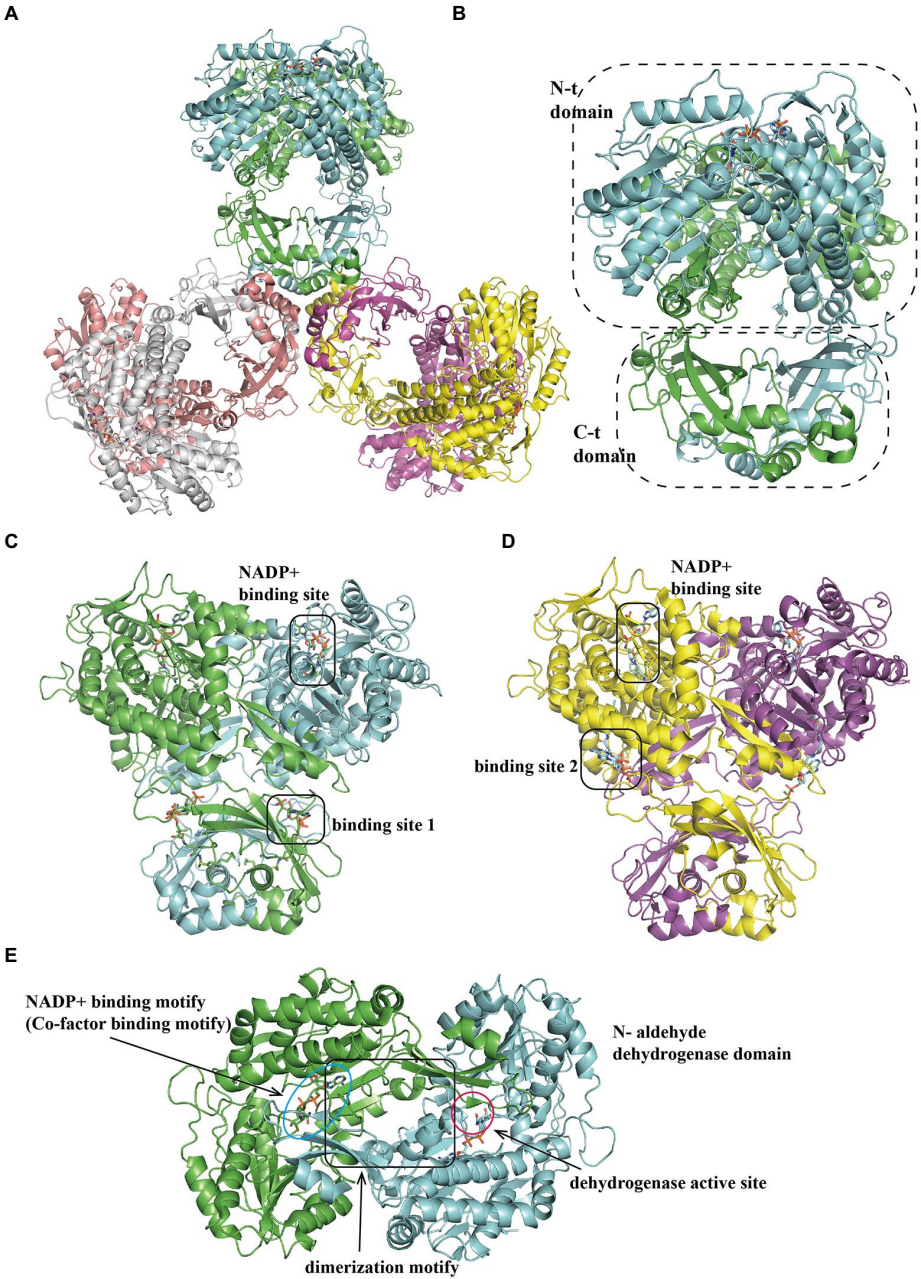
Overall structure of PaaZ. **(A)** The overall structure of the tri-lobed architecture hexamer EcoPaaZ (PDB:6JQM) is shown in different colors for each chain. The architecture unit PaaZ dimer reveals an N-terminal aldehyde dehydrogenase domain and the C-terminal R-specific hotdog fold hydratase domain, circled with a dotted line. For clarity, in the substrate binding domain, the rotated views from **(A)** are shown in **(B)** with OCoA (PDB:6JQN) and with CCoA (PDB:6JQO; **C**) in different binding site 1 or 2, respectively. The NADP^+^-binding site is the same in **(B)** and **(C)**. **(D)** The N-terminal dehydrogenase domain can be divided into three sub-domains: co-factor binding, catalytic and dimerization motif, shown with lines and annotation, respectively.

The tri-lobed hexamer PaaZ was maintained by the inner core formed by the C-hydratase domain of three dimers. The hydratase domain consists of a mixture of α-helices and β-strands, where the α-helices are involved in dimer oligomerization (Figures 6A,C,D). The dehydrogenase domain was further divided into three regions: co-factor binding, catalytic, and dimerization motifs (Figure 6B). Previous studies have shown that the product of the hydratase domain is a substrate for dehydrogenase (Teufel et al., 2011). A recent study of PaaZ structures with open-ring mimics, octanoyl CoA (Figure 6C) and crotonyl-CoA (Figure 6D), provides a possible model for substrate transfer. The bifunctional enzyme PaaZ presents a positively charged surface at the entrance of the hydratase and dehydrogenase domains, which is complementary to the negatively charged coenzyme A in the substrate. Through this tunnel, by electrostatic pivoting of the CoA part, the key intermediate can transfer from one active site to another internally without being released into the bulk solvent (Sathyanarayanan et al., 2019).

Interestingly, PaaZ contains only an aldehyde dehydrogenase domain in several phenylacetate-degrading organisms such as *Aromatoleum aromaticum*. A hotdog-fold hydratase encoded by a gene outside the *paa* operon has been identified to perform the compound IV hydrolysis, capable of hydrating crotonyl-CoA with high activity, which could replace the missing hydratase function in PaaZ (Teufel et al., 2011).

### Toxic epoxide control

#### Reversible regulation in PAA pathway

Multiple epoxides produced during the *Paa* catabolic pathway are toxic to cells, and several mechanisms have evolved in bacteria to control these toxic metabolites.

The first epoxides generated in the PAA pathway are mediated by the PaaABCE complex epoxidation of the aromatic ring. The oxygenase complex itself can also perform reverse reaction-deoxygenation to yield the compound II (Figure 1), as discussed above. The PaaABCE complex assists in excess epoxide removal when the inadequate processing by downstream enzymes, PaaG and PaaZ, leads to substrate accumulation (Teufel et al., 2012).

To protect PaaABCE from overloading and avoiding subsequent toxic accumulation, PaaI catalyzes a reverse reaction to the compound II ligase PaaK, removing excess the compound II (Teufel et al., 2012). PaaI exhibited a narrow substrate specificity to the compound II in relevant pathway intermediates, with an activity of 1.6 U mg^−1^ measured at 22°C (Teufel et al., 2012). The EcoPaaI *K_m_* was 9.6 μM and *K_cat_* was 4.1·10^−1^ s^−1^ under optimum conditions of 25°C and pH 7.5. And for AevPaaI, the *K_m_* and *K_cat_* for II was 9.6 μM and 4.1·10^−1^ s^−1^, respectively (Song et al., 2006). In another study, enzyme kinetic analysis revealed that SphPaaI shows high activity against decanoyl for the compound II with *K_m_* of 90 μM, *K_cat_* of 6.5 s^−1^, and specificity constant of 7.2 × 10^4^ m^−1^ s^−1^, while a higher activity was observed for medium-chain fatty acyl-CoA substrates (decanoyl/C10-CoA) with corresponding values of 183 μM, 32.8 s^−1^, and 1.8 × 10^5^ m^−1^ s^−1^, respectively (Khandokar et al., 2016).

**Figure 7:**
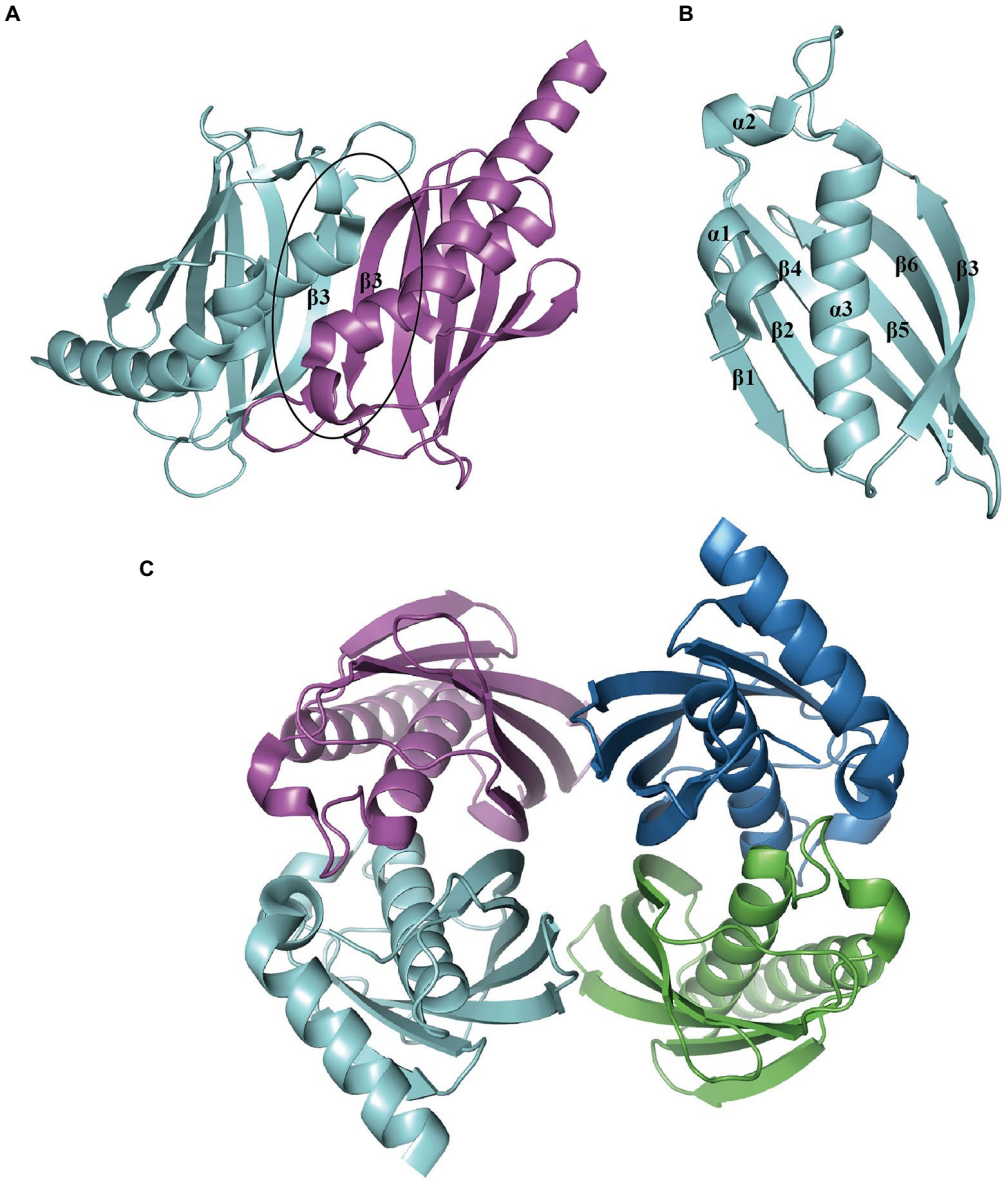
Overall structure of PaaI. **(A)** The PaaI reveals a hotdog fold shape with a core α-helix (α3) enveloped by a six-strand β-sheet (β1-β6). **(B)** The architecture unit PaaI dimer is formed by the interaction between β-sheets in each monomer. **(C)** The overall structure of tetramer TthPaaI (PDB:1J1Y) is shown in different colors for each chain.

The representative hotdog-fold structure for PaaI consists of a core α-helix enveloped by an aβ-sheet formed by six strands on one side (Figure 7A). The native molecule works as a homotetramer; the β-sheet from two monomers stack together to form a continuous 12-stranded antiparallel β-sheet (Figure 7B), which associates back-to-back with a second dimer (Figure 7C). The core α-helix also participates in the oligomerization of dimers. The ligand acyl-CoA is located on the dimer surface and binds to the acyl-thioester moieties (Song et al., 2006).

#### Toxic epoxide cleavage

**Figure 8:**
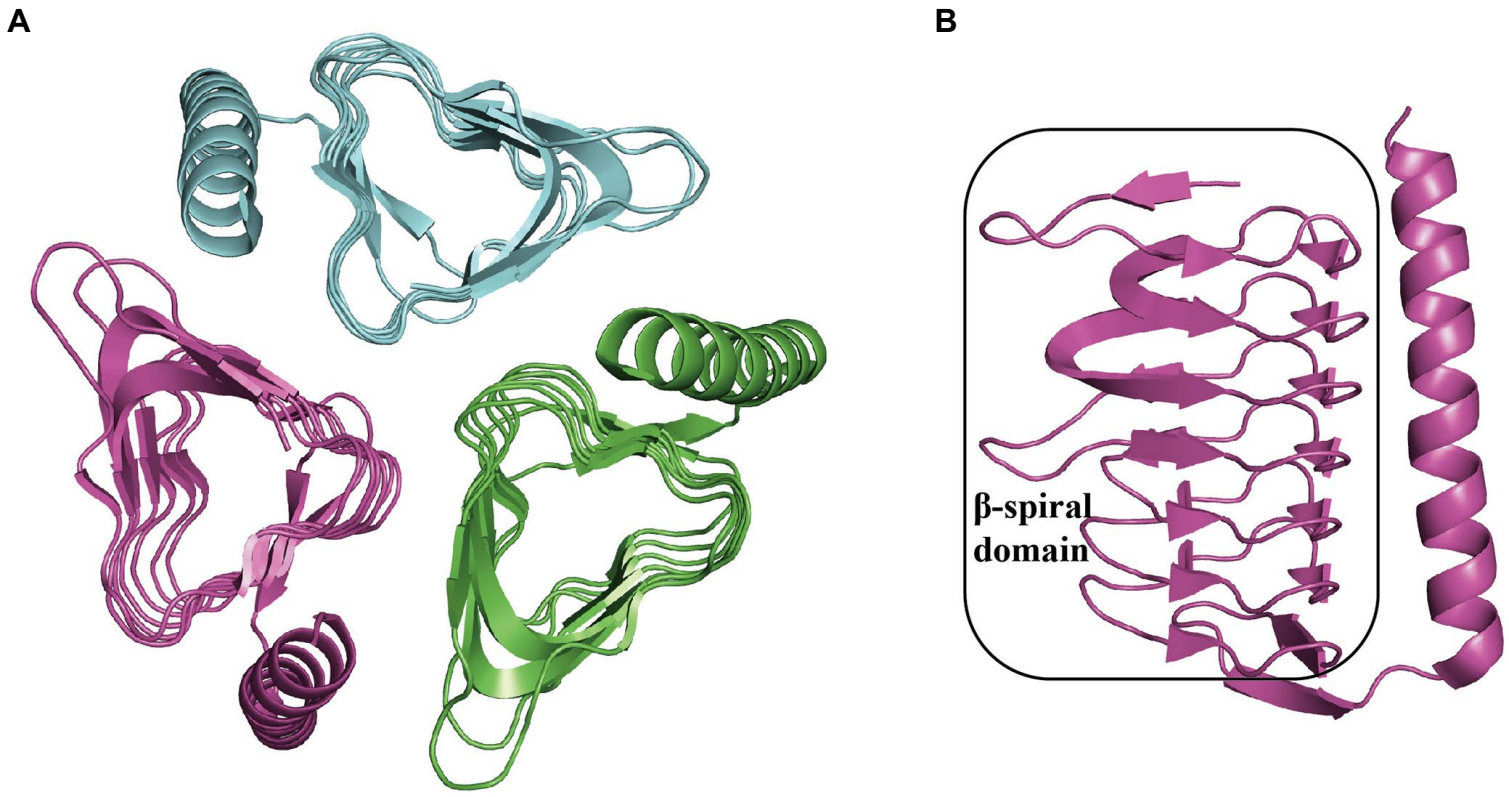
Overall structure in PaaY. **(A)** The overall structure of trimer GkaPaaY (PDB:3VNP) is shown with a different color for each chain. **(B)** The PaaY consists of a large β-spiral containing one C-terminal α-helix connected by an extraβ-strand.

Thioesterase PaaY is necessary for the efficient degradation of PAA in *E. coli.* It serves as a regulatory protein in the PAA pathway, which could specifically hydrolyze the compound XI, an inhibitor to PaaZ when NADP^+^ deficiency occurs and labile aldehyde oxidation is impaired in the cell, with an activity of 7.6 U mg^−1^ and *K_m_* 35 μM at 22°C (Teufel et al., 2011, 2012) The disruption of EcoPaaY and PpuPaaY showed no effect on PAA catabolism (Ferrández et al., 1998; Olivera et al., 1998), but revealed an obvious lag phase in growth and morphological changes when using PAA as the sole carbon source (Fernández et al., 2014). EcoPaaY associates with trimers containing Ca^2+^ and Zn^2+^ ions and shows a wider substrate range of CoA derivatives. The optimal reaction conditions for EcoPaaY were pH 8.0 and 45°C; in addition, thioesterase activity increased in the presence of Co^2+^ whereas Cu^2+^, Mn^2+^, and Ni^2+^ had an inhibitory effect (Teufel et al., 2010; Fernández et al., 2014). A homolog of PaaY with 33% similarity from *Geobacillus kaustophilus* was structurally characterized (PDB:3VNP) as a homotrimer (Figure 8A; Paper unpublished). A new function of PaaY and its connection with the regulation of the *paa* gene cluster need to be investigated.

### Late steps

#### ß-oxidation leads to open-ring intermediate cleavage

The crucial step in the late part of *Paa* pathway is the catalysis of long-chain intermediate fission, mediated by thiolase PaaJ. PaaJ serves as a β-ketoadipyl-CoA thiolase to clear the C8-intermediate formed by PaaZ, finally forming a C6-intermediate for subsequent processes.

**Figure 9:**
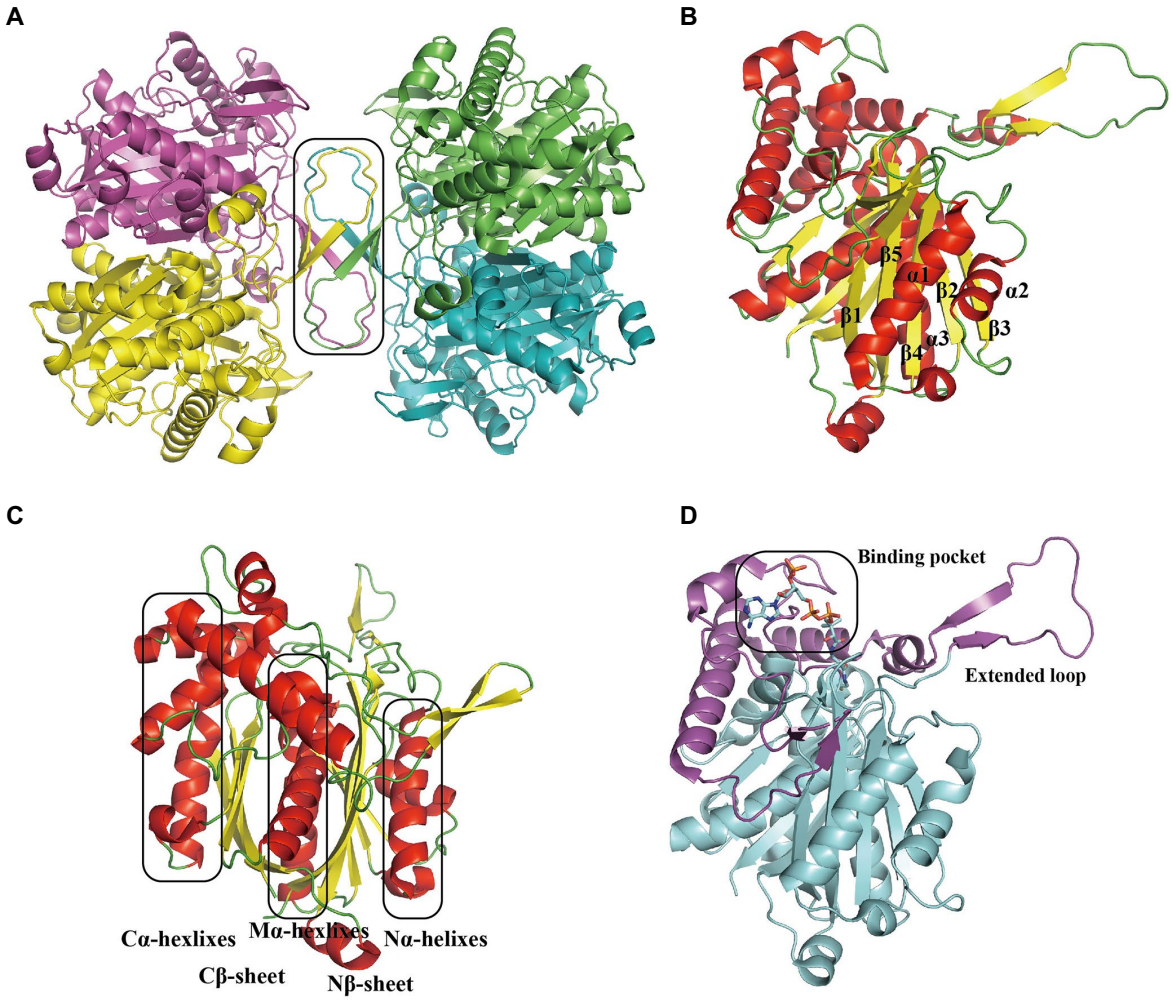
Overall structure of PaaJ. **(A)** The overall structure of TthPaaJ (PDB: 1ULQ) reveals a tetramer shown in a different color for each chain with two lobes and a central β-barrel composed of four monomers circled with a solid line. **(B)** The PaaJ monomer is colored by secondary structure, red, yellow, and green for the α-helix, β-strands, and loop, respectively. The N-terminal and C-terminal region reveals a similar βαβαβαβ topology (the secondary structure elements in the N-terminal are annotated). **(C)** The rotated view in **(B)**, reveals a sandwich architecture where the N-terminal and C-terminal β-sheet are covered by two-layer α-helices. N, C and M for N-terminal, C-terminal and medial, respectively. **(D)** The extended loop domain between Nβ4 and Nβ5 in the N-terminal β-sheet is involved in CoA binding. The loop is shown in magenta while the rest is in cyan, and the binding pockets are circled with a solid line.

TthPaaJ (PDB:1ULQ) belongs to the thiolase superfamily; the overall structure of TthPaaJ reveals a two-lobe-tetramer linked by a central β-barrel composed of four monomers (Figure 9A). The N- and C-termini in the monomer share a similar “βαβαβαβ” topology, in which the β-strands fold into a five-stranded or four-stranded mixed-sheet sandwich covered by α-helices (Pfündel, 2021; Figure 9B). A loop domain between Nβ4 and Nβ5 extending from the N-terminal β-sheet is mainly composed of α-helices and folds on top of the thiolase core domain, involving CoA in binding and determining substrate specificity (Kiema et al., 2014; Figures 9C,D). In addition, this loop is crucial for the function of human mitochondrial 3-ketoacyl-CoA thiolase, a homolog of PaaJ (PDB:4CJ2; Kiema et al., 2014; Figure 9D).

#### Unsaturated thioester isomerization and hydration

The S-specific hydratase PaaF, together with PaaG, belong to the crotonase-fold superfamily. In the next two steps, PaaG isomerizes the unsaturated thioester and the PaaF hydrated compound, finally generating the compound IX (Figure 1). The features of the PaaG isomerase are discussed above. Previous studies using mass spectrometry have shown that purified PaaF can catalyze the reversible conversion of substrates between the compound VIII and the compound IX. In addition to PaaABCE, PaaF-PaaG was the only stable complex in the late steps of the PAA pathway, which may be an evolutionary adaptation to speed up subsequent reactions in the pathway (Grishin et al., 2012). The PaaFG complex contained a stack of four homotrimeric discs assembled by two PaaF discs in the center sandwiched between PaaG discs at each end (Figure 5C). The PaaF monomer exhibits a crotonase fold that is highly similar to that of PaaG (Figure 5D), and the active sites are located on the external surfaces of the disc structure, similarly to those of PaaG (Figure 5E; Grishin et al., 2012).

#### Oxidation dehydrogenation and cleavage

**Figure 10:**
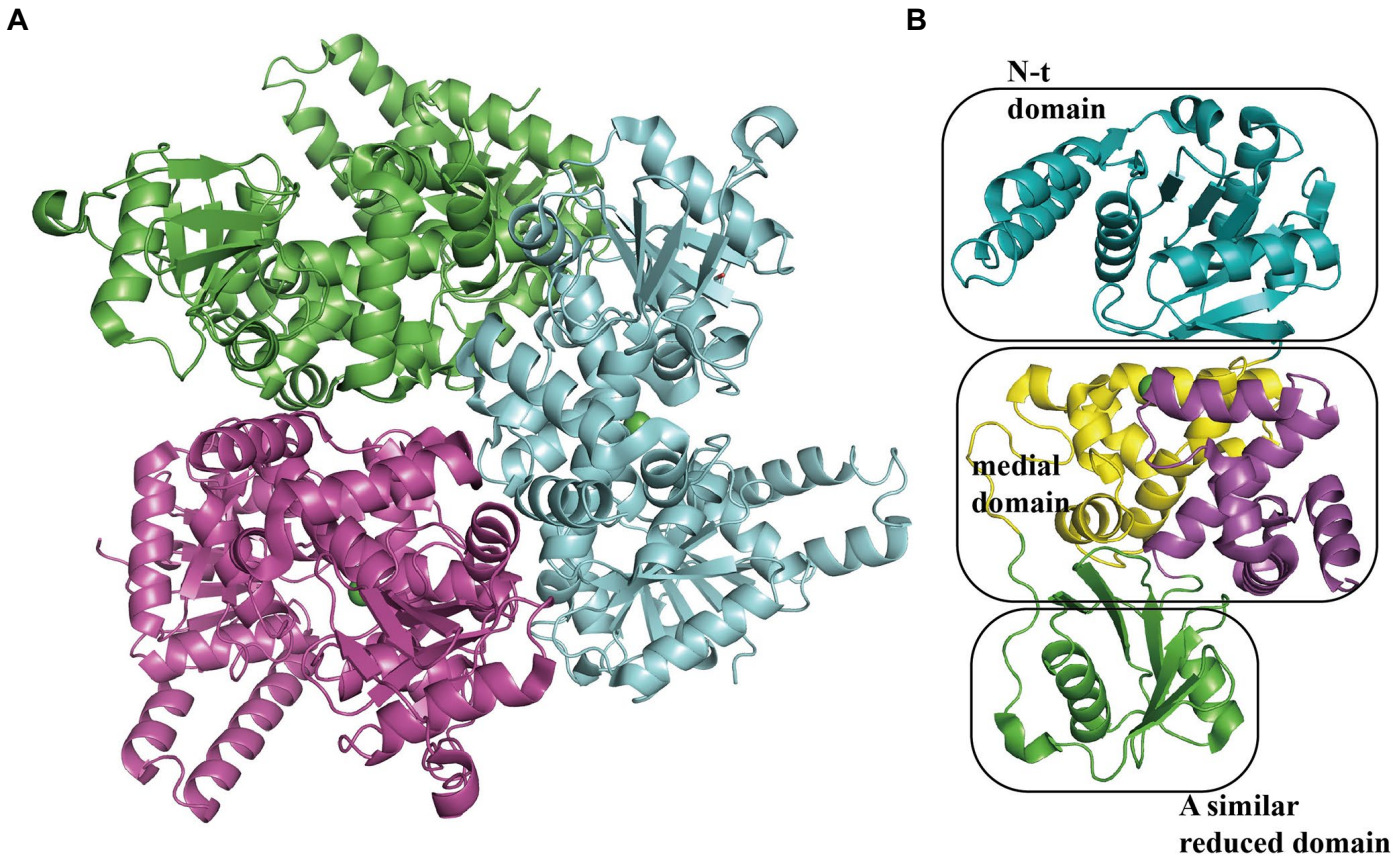
Overall structure of PaaH. **(A)** The overall structure of the EcoPaaH trimer (PDB:3MOG). The PaaH trimer reveals a central symmetric structure in space, shown in green, magenta, and teal by chains. **(B)** The PaaH monomer is constructed from three regions, N-terminal domain, and a reduced domain (mainly consisting of a β-sheet surrounded by α-helices) in teal and green, respectively. In addition, a medial region consists of two discontinuous parts sharing a similar five-helixes structure which could be seen as a ‘dimer’ conformation, shown in yellow and magenta in each part, respectively.

The 3-hydroxyadipyl-CoA dehydrogenase PaaH oxidizes substrates the compound IX to X, depending on NAD+ (Teufel et al., 2010). The EcoPaaH trimer (PDB:3MOG) has been observed (Figure 10A; Paper unpublished). The monomer revealed a sandwich shape with three regions, a medial region located at the interface of the trimer packaged by two similar domains (N-terminal domain and a reduced domain, mainly consisting of a β-sheet surrounded by α-helices) on both sides. The medial region consists of two similar but discontinuous parts, both mainly formed by five α-helices (Figure 10B). Finally, the last step of the pathway, cleaving of X is also catalyzed by PaaJ. Using an HPLC chromatogram measuring end-products *in vitro*, EcoPaaJ was confirmed to generate acetyl-CoA and succinyl-CoA due to thiolytic fission of β-ketoadipyl-CoA (Nogales et al., 2007).

#### Regulation of phenylacetate catabolic pathway

GntR-type and TetR-type are two existing systems that regulate PAA catabolism, both in response to II as an inducer. In *E. coli*, *P. putida*, and other *Pseudomonas* species, PaaX is a representative GntR-type regulatory protein (Ferrández et al., 2000; García et al., 2000; del Peso-Santos et al., 2006). Conversely, PaaR is a TetR-type protein present in *T. thermophilus*, *C. glutamicum*, and *B. cenocepacia* (Hamlin et al., 2009; Sakamoto et al., 2011; Chen et al., 2012).

#### GntR-type regulator

**Figure 11:**
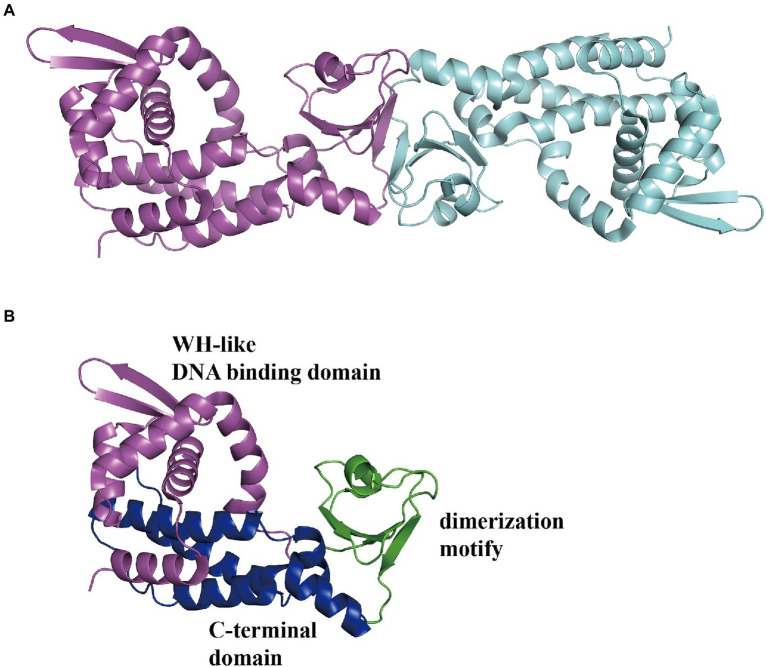
Overall structure of PaaX. **(A)** The overall structure of dimer Jccs1PaaX (PDB:3 L09) is shown in a different color for each chain. **(B)** The PaaX could be divided into three regions as an N-terminal Winged helix-like DNA-binding domain, a dimerization motif, and a C-terminal extended domain consisting of five α-helices, shown in magenta, green, and blue, respectively.

In *E. coli*, the *Pz* and *Pa* promoters control two divergently transcribed operons, *paaZ* and *paaABCDEFGHIJK*, respectively, which are negatively regulated by PaaX and are transcribed on an adjacent *Px* operon (Ferrández et al., 2000). The *Px* promoter in charge of the *paaXY* operon expressing the *paaX* regulatory gene and thioesterase PaaY is repressed by its own product PaaX, based on the steric hindrance of RNAP binding to the *Px* promoter (Fernández et al., 2014). In addition, II could specifically inhibit the binding of PaaX to the target sequences of *Pa* or *Pz*, confirming the first intermediate the compound II in the PAA pathway as the true inducer, but not PAA (Ferrández et al., 1998; Fernández et al., 2014). Jccs1PaaX (PDB 3 L09; Figure 11A) revealed an N-terminal winged helix-turn-helix (wHTH) DNA-binding domain, a dimerization motif, and a C-terminal extended domain (Figure 11B). The binding base sequence of EcoPaaX is TGATTC(N27)GAATCA (Kim et al., 2004a,b), and a similar sequence was found in Py2PaaX (del Peso-Santos et al., 2006). The specific C-terminus binds II to activate the N-terminal domain (Ferrández et al., 2000). PaaX competes with RNA polymerase to bind to the regulatory *Px* and *Pz* promoters, but the mechanism of binding to the *Pa* promoter is different (Fernández et al., 2014). The complex structure of PaaX and its binding operator bases have not yet been elucidated.

#### TetR-type regulator

In *B. cenocepacia*, PA-related genes are located in three separate clusters: *paaABCDE*, *paaFZJGIJK_1_*, and *paaHK_2_*. A regulatory gene, PaaR, was identified downstream of the *paaABCDE* gene cluster oriented in the same direction, but in a separate transcriptional unit different from *paaABCDE* (Hamlin et al., 2009). The N-terminal region of BcePaaR shows high similarity to the TetR-type regulator of the multi-drug efflux pump EcoAcrR (Hamlin et al., 2009). Through insertional mutagenesis, PaaR was confirmed to be a negative regulator of the promoters of P*paaA*, P*paaH*, and P*paaZ* (Hamlin et al., 2009). In addition, a 15 bp inverted repeat (IR) sequence site serving as the operator site in P*paaA*, P*paaH,* and P*paaZ* (ACCGACCGGTCGGTT in P*paaA* and P*paaZ,* ACCAACCGGTCGGTT in P*paaH*) was validated by constructing eGFP translational fusion plasmids and reporter activity measurements (Hamlin et al., 2009). In a subsequent study, electrophoretic mobility shift assay (EMSA) was used to confirm the binding capacity between PaaR and target sequences (Yudistira et al., 2011). In addition, the compound II, but not the compound I, can dissociate PaaR from the target promoter regions (Yudistira et al., 2011).

Another study reported the same conclusion regarding the features of *Thermus thermophilus* TthPaaR (Sakamoto et al., 2011). Using the BIAcore biosensor assay, TthPaaR was confirmed to bind two DNAs with a consensus sequence of CNAACGNNCGTTNG and similar values of association rate constant, dissociation rate constant, and dissociation constant at 9.3 × 10^5^ M^−1^ s^−1^, 1.0 × 10^−3^ s^−1^, and 1.1 nM, and 9.8 × 10^5^ M^−1^ s^−1^, 0.9 × 10^−3^ s^−1^, and 0.9 nM, for TthPaaR binding site 1 and 2, respectively (Sakamoto et al., 2011). The DNA binding ability of PaaR decreased in a concentration-dependent manner in the compound II. In addition, ITC results revealed that the compound II binding with PaaR was *K_d_* at 4.1 × 10^7^ M^−1^, and the number of binding sites on PaaR was approximately 1.3 (Sakamoto et al., 2011). The putative structure of TthPaaR revealed a typical TetR family member with a predicted N-terminal DNA-binding domain consisting of a helix-turn-helix motif with a positively charged surface (Sakamoto et al., 2011).

## Significance of the PAA pathway

The well-studied PAA pathway, as the final degradation mechanism for multiple aromatic hydrocarbons, has been widely applied to specific products generated *in vivo via* the genetic engineering technology (Chen et al., 2018; Li et al., 2019). Additionally, our knowledge of its native role in bacteria remains limited. Here, we concluded that the PAA pathway plays a role in bacterial pathogenicity and antibiotic resistance.

### Crosslink between the PAA pathway and antibiotic resistance

The PAA pathway is closely linked to antibiotic resistance. The side reaction intermediate products XI of the PAA catabolism is the proposed universal precursor for tropone natural products and their derivatives (Teufel et al., 2011; Brock et al., 2014; Duan et al., 2020, 2021), among which tropodithietic acid (TDA) is a broad-spectrum antimicrobial compound (Geng et al., 2008; Henriksen et al., 2022). TDA also works as a signaling molecule, which influences phenotypic traits like motility and biofilm formation, and gene expression of other bacteria and antibiotic production in the producer (Geng et al., 2008; Beyersmann et al., 2017; Duan et al., 2020).

Penicillin G acylase (PGA), which hydrolyzes penicillin G to 6-aminopenicillanic acid (6-APA) and PAA, is thought to serve as a scavenger of many different natural esters and amides of PAA or its derivatives in EcoPaaX has been shown to specifically bind the *Ppga* promoter This binding effect could be inhibited by PA-CoA, as discussed above (Galán et al., 2004; Kim et al., 2004a,b). Despite PGA showing no functions in bacterial antibiotic resistance, these findings lay the foundation for the connection between the PAA pathway and antibiotic resistance.

In *B. cenocepacia*, global gene expression analysis suggested that multiple enzymes in the PAA degradation pathway are upregulated in response to meropenem exposure, indicating a potential connection between PAA and antibiotic resistance (Sass et al., 2011). Coincidentally, the PAA degradation pathway in *A. baumannii* is significantly upregulated in response to ceftazidime (Alkasir et al., 2018) and lethal concentrations of ciprofloxacin (Kashyap et al., 2021), suggesting that the PAA pathway is a suitable pathogen control target. Furthermore, *paa* genes were found to be downregulated in a ∆*adeIJK* mutant of *A. baumannii*, with AdeIJK efflux as a broad-spectrum pump, especially for amphiphilic compounds (Damier-Piolle et al., 2008; Leus et al., 2020).

A recent study provided more evidence that treatment with antibiotics at a subinhibitory concentration led to an approximately 7-fold increase in the expression of *paaA* and *paaB* to impact intracellular PAA levels in *A. baumannii* (Hooppaw et al., 2022). They also reported that PAA catabolism is important for *A. baumannii* in multiple antibiotic stress conditions, especially in the presence of cytoplasmic targets such as ciprofloxacin, erythromycin, and tetracycline, where the biofilm formation ability is repressed in the WT strain but not impacted in a deletion mutant strain of *paaB* (Hooppaw et al., 2022).

### Relationship between the PAA pathway and bacterial pathogenicity

High levels of PAA inhibit the pathogenicity of the fungus *Rhizoctonia solani*; 7.5 mM PAA in the growth medium reduced the biomass to 50% (Bartz et al., 2012). In *A. baumannii*, a deletion mutant of paaE in a mouse septicemia model showed significantly attenuated virulence (Cerqueira et al., 2014). PA-CoA attenuated CepIR-regulated virulence in *B. cenocepacia*, suggesting that a metabolic signal can activate virulence in the absence of QS signaling molecules (Lightly et al., 2019).

#### Relation between the PAA pathway and biofilm and H_2_O_2_ tolerance

Biofilms are formed by *A. baumannii* on abiotic and biotic surfaces to survive in human serum and infection, resistance to desiccation stress, and starvation in the nosocomial environment (Zeidler and Muller 2019). A recent study on the PAA pathway under antibiotic treatment in *A. baumannii* suggested that PAA could induce biofilm formation depending on the expression of Csu, a pili protein, which is one of the main determinants of biofilm formation (Hooppaw et al., 2022). The exogenous addition of PAA can reverse the inhibition of Csu during antibiotic treatment (Hooppaw et al., 2022).

Previous studies have demonstrated a connection between the PAA pathway and oxidative stress. Expression of the *paa* operon was downregulated in a deletion mutant *A. baumannii* strain of MumR, a transcriptional regulator involved in Mn^2+^ uptake and H_2_O_2_ tolerance (Green et al., 2020). A deletion mutant Δ*paaJKXYI* in the PAA pathway, rather than WT, was more susceptible to the lethal effects of H_2_O_2_, but not restricted to growth, which may occur in high concentrations of H_2_O_2_ (Green et al., 2020).

#### Intrinsic interaction between the PAA pathway and quorum sensing

The quorum sensing (QS) system that works on the production and detection of signaling molecules is vital in bacterial intercellular communication; further, this system can bind the transcriptional regulator to activate the expression of virulence factors in several opportunistic pathogens. The relationship between the PAA pathway and bacterial pathogenicity has been well described in the opportunistic pathogen *Burkholderia cenocepacia*, which establishes persistent infections in humans the genetic diseases, cystic fibrosis and pulmonary cystic fibrosis (Law et al., 2008; Imolorhe and Cardona, 2011; Pribytkova et al., 2014; Lightly et al., 2019).

In insertional mutant *B. cenocepacia* strains, researchers first confirmed that PaaA and PaaE are important for infection through displaying attenuated pathogenicity in *Caenorhabditis elegans* without defects in growth and colonization in the host (Law et al., 2008). Subsequent studies have provided compelling evidence connecting the PAA pathway and QS system. Researchers have found that exogenous addition of PAA attenuates the pathogenicity of the ΔpaaABCDE strain, and further studies have demonstrated that the signal molecules in QS are inhibited, which is vital for virulence factor expression. Thus, PAA can participate in the QS-regulated pathogenic responses. Meanwhile, QS (CepIR)-regulated virulence traits, and *cepI* and *cepR* promoter activity were downregulated in the ΔpaaABCDE strain (Pribytkova et al., 2014). Taken together, these findings highlight a direct connection between PAA metabolism and QS-regulated pathogenic responses.

However, a recent study reported that the PaaK knockout mutant strain is more virulent, which is in contrast to the less virulent ΔpaaABCDE strain. By constructing deletions of the *cepI* and *cepR* genes in the PAA pathway mutant backgrounds, they suggested that there is an alternative signaling pathway to activate virulence in theΔpaaK1 paaK2 ΔcepR mutant, in which PAA-CoA or a derivative, but not PAA, is the central molecule (Lightly et al., 2019). Further studies are needed to uncover the complex internal regulatory mechanisms of the PAA pathway and the QS system.

#### Intrinsic interaction between the PAA pathway and host immune

In *A. baumannii*, the entire *paa* operon is controlled by GacSR, a two-component regulatory system sensor kinase, which is also a global virulence regulator responsible for inducing the expression of 674 genes, including biofilm formation and virulence-related genes, responsible for toxicity (Cerqueira et al., 2014). PAA was characterized from the culture supernatants of attenuated cytotoxicity *Pseudomonas aeruginosa* at high cell density, and PAA can downregulate the expression of virulence-related genes such as T3SS and its related regulatory genes (Wang et al., 2013). In a zebrafish infection model, PAA serves as a neutrophil chemoattractant, and the PAA pathway is crucial in tissue responses to acute infection, whose inhibition contributes to neutrophil response and invader clearance (Cerqueira et al., 2014; Bhuiyan et al., 2016; Kröger et al., 2016). Thus, the PAA pathway is involved in immune evasion and disease progression during the interaction between *A. baumannii* and its host.

Taken together, the PAA pathway shows the ability to crosstalk among multiple systems involved in bacterial pathogenicity and is a potential target in infection treatment. More work is needed to determine the regulatory relationship of the PAA pathway and measure its potential in restricting microbial infection.

## Author contributions

MJ, WH, ZO, QS, and YW wrote the manuscript. WH made all the structural figures under the supervision of YW. All authors contributed to the article and approved the submitted version.

## Funding

This work was supported by the National Natural Science Foundation of China (Nos. 31870132, 82072237, and 82102402), Shaanxi Province Natural Science Funding (No. 2021JQ-381), and Institutional Foundation of the First Affiliated Hospital of Xi’an Jiaotong University.

## Conflict of interest

The authors declare that the research was conducted in the absence of any commercial or financial relationships that could be construed as a potential conflict of interest.

## Publisher’s note

All claims expressed in this article are solely those of the authors and do not necessarily represent those of their affiliated organizations, or those of the publisher, the editors and the reviewers. Any product that may be evaluated in this article, or claim that may be made by its manufacturer, is not guaranteed or endorsed by the publisher.

## Abbreviations

BTEX – benzene, toluene, ethylbenzene, and xylene
CoA – coenzyme A
LC–MS –
NBT – nitroblue tetrazolium
PAA – phenylacetic acid pathway
PAHs – polycyclic aromatic hydrocarbons
PCBs – polychlorinated biphenyls
PGA – penicillin G acylase
QS – quorum sensing
TDT – tropodithietic acid
